# Resolvin D1 Attenuates Doxorubicin-Induced Cardiotoxicity by Inhibiting Inflammation, Oxidative and Endoplasmic Reticulum Stress

**DOI:** 10.3389/fphar.2021.749899

**Published:** 2022-01-05

**Authors:** Menglong Wang, Jishou Zhang, Mengmeng Zhao, Jianfang Liu, Jing Ye, Yao Xu, Zhen Wang, Di Ye, Dan Li, Jun Wan

**Affiliations:** ^1^ Department of Cardiology, Renmin Hospital of Wuhan University, Wuhan, China; ^2^ Cardiovascular Research Institute, Wuhan University, Wuhan, China; ^3^ Hubei Key Laboratory of Cardiology, Wuhan, China; ^4^ Department of Pediatrics, Renmin Hospital of Wuhan University, Wuhan, China

**Keywords:** resolvin D1, doxorubicin, cardiotoxicity, oxidative stress, apopstosis

## Abstract

Resolvin D1 (RvD1) is a lipid mediator that promotes resolution of inflammation. However, the function of RvD1 in doxorubicin- (Dox-) induced cardiotoxicity remains to be clarified. This study aimed to investigate whether RvD1 could attenuate Dox-induced cardiac injury. The mice were divided into three groups: control, Dox (20 mg/kg, once, intraperitoneally), and Dox + RvD1. RvD1 (2.5 μg/kg, intraperitoneally) was injected daily for 5 days. Echocardiography was performed to evaluate the cardiac function, and the heart tissue and serum samples were collected for further analyses. The results showed that RvD1 attenuated the decreased ratio of heart weight/body weight and heart weight/tibia length, the increased level of creatine kinase and activity of lactate dehydrogenase after Dox treatment. RvD1 improved the ejection fraction and fractional shortening of left ventricular and attenuated the severity of apoptosis induced by Dox. As for the underlying pathways, the results showed that RvD1 reduced the expression of IL-1 and IL-6, and attenuated the phosphorylation of P65 in cardiac tissue. RvD1 attenuated the oxidative stress induced by Dox, as demonstrated by the attenuated levels of superoxide dismutase, glutathione, and malondialdehyde, decreased expression of Nox-2 and Nox-4 and increased expression of Nrf-2 and HO-1. In addition, RvD1 also inhibited the endoplasmic reticulum stress induced by Dox. These results indicate the potential therapeutic benefits of RvD1 in Dox-induced cardiotoxicity in mice, and the mechanism may be related to the attenuated inflammation, oxidative stress and endoplasmic reticulum stress.

## Introduction

Doxorubicin (Dox), an anthracycline-based chemotherapeutic drug, is routinely used in the treatment of a wide variety of cancers, including breast, ovarian, bladder, lung, thyroid, and stomach cancer (Buzdar et al., 1985). However, treatment with Dox has been reported to cause dose-dependent cardiac toxicity and heart failure (Lefrak et al., 1973). Dox-induced cardiotoxicity seriously impairs the quality of life and life expectancy of patients with cancer. Several studies have shown that oxidative stress and apoptosis of cardiomyocytes are associated with Dox-induced cardiotoxicity (Kalivendi et al., 2001). However, there are currently no effective drugs to prevent and treat the cardiotoxicity caused by Dox.

Resolvin D1 (RvD1) is a specialized pro-resolving lipid mediator, mainly derived from docosahexaenoic acid (DHA). RvD1 reduces excessive polymorphonuclear neutrophil infiltration and transmigration, promoting resolution of inflammation (Abdolmaleki et al., 2020). In addition, RvD1 reduces tissue damage by attenuating oxidative stress and apoptosis (Lee and Surh, 2013; Saito et al., 2018). RvD1 has a protective role in Dox-induced nephropathy (Zhang et al., 2013). The protective effect of RvD1 has also been reported in cardiovascular diseases, including myocardial injury, neointimal hyperplasia, and abdominal aortic aneurysm (Miyahara et al., 2013; Kain et al., 2015; Spinosa et al., 2018). Recently, RvD1 was reported to alleviate angiotensin II-induced hypertension and cardiac remodeling via blocking Ang II signaling and attenuating inflammation (Olivares-Silva et al., 2021; Salas-Hernández et al., 2021).

However, the function of RvD1 in Dox-induced cardiovascular injury remains unclear. The objective of our study was to determine the effect of RvD1 supplementation on Dox-induced myocardial damage and to identify the underlying mechanism, which may provide novel insights for the prevention and/or treatment of Dox-induced cardiotoxicity.

## Materials and Methods

### Animals

Experimental mice were treated in accordance with the National Institute of Health Guidelines for the Care and Use of Laboratory Animals, and study was approved by the Ethics Committee for Animal Research of the Wuhan University (Institutional Animal Care and Use Committee Issue No.20181215). C57BL/6 male mice (6–8 weeks old, 21.5–22.5 g) were obtained from Vital River Experimental Animal Technology Co. Ltd. (Beijing, China). The mice were maintained in a standard laboratory at the Cardiovascular Research Institute of Wuhan University, and were housed in standard humidity/temperature-controlled environment (70% relative humidity, 22°C) in a light-controlled room (12/12 h light/dark cycle) with access to sterile rodent food and water. All the mice were individually caged. The mice were used for the experiment after acclimatization to the housing environment for 2 weeks. The mice were randomly divided into three groups: control (CTRL; n = 10), Dox (n = 15), and Dox + RvD1 (n = 10). The control group received only sterile saline. Mice in the Dox group were treated with Dox (20 mg/kg) once intraperitoneally (i.p.) (Wang et al., 2018; Zhang et al., 2020). Mice in the Dox + RvD1 group were treated with RvD1 (2.5 μg/kg, i.p.) 30 min before Dox administration and every day thereafter for the duration of the experiment. Both Dox and RvD1 were dissolved in 0.9% sterile saline. All mice were observed and weighed daily. The mice were sacrificed after 5 days of Dox treatment. The heart weight of mice was collected for the ratio of heart weight (HW)/body weight (BW) and HW/tibia length (TL). The left cardiac tissues were collected for detailed analyses.

### Echocardiography

Echocardiography was performed on anesthetized (1.5–2% isoflurane) mice using a Mylab30CV ultrasound (Biosound Esaote, Inc.) equipped with a 10 MHz linear array ultrasound transducer. We defined end-systole and end-diastole as the phases in which the left ventricular (LV) end-diastolic diameter (LVEDd) and LV end-systolic diameter (LVEDs) were obtained. LV ejection fraction (LVEF) and LV fractional shortening (LVFS) were also analyzed via LV M-mode tracing with a sweep speed of 50 mm/s at the midpapillary muscle level.

### Cardiomyocyte Injury Evaluation

The activity of lactate dehydrogenase (LDH), the concentrations of myocardial-bound creatine kinase (CK-MB) and cardiac troponinwere I (cTnI) assessed as indexes of cardiomyocyte injury. Both LDH activity, CK-MB levels and cTnI levels in serum were detected using kits (all purchased from Nanjing Jiancheng Bioengineering Institute, China) according to the manufacturer’s instructions and as described in our previous study (Ye et al., 2018).

### Oxidative Stress Evaluation

At the end of the experiment, the cardiac tissues were removed and washed in ice-cold phosphate-buffered saline. The cardiac tissues (30 mg) were added to 300 μL of phosphate-buffered saline, ground into homogenates, and centrifuged at 3,000 rpm at 4°C for 15 min to collect the supernatant. The activities of superoxide dismutase 1 (SOD1) and the level of malondialdehyde (MDA) and glutathione (GSH) were detected by commercially available kits purchased from Nanjing Jiancheng Bioengineering Institute (Nanjing, China) following the manufacturer’s instructions.

### Histological Analysis

Histological analysis was performed as described in our previous study (Ye et al., 2020a). Cardiac tissues were fixed with 4% paraformaldehyde for 5 days. Then, the tissues were embedded in paraffin and sliced into 4–5 μm sections and mounted onto slides. Cardiomyocyte vacuolization was analyzed by hematoxylin and eosin (H&E) staining using a commercially available kit (Millipore) and then visualized by light microscopy. Sections were also subjected to immunofluorescence staining. the sections were autoclaved for antigen retrieval and then blocked with 10% goat serumfor 10min. Next, the sections were incubatedwith primary antibodies against Phospho-NF-κB p65 (Abcam, Cambridge, United Kingdom) overnight at 4°C. The sections were rinsed with PBS for 20 min before incubating with two different IRDye^®^ 800CW conjugated secondary antibodies for 60 min and subsequently counterstained with the SlowFade Gold antifade reagent containing DAPI. All the figures were captured with fluorescence microscope, and Image Pro Plus 6.0 (Media Cybernetics, Bethesda, MD, United States) was used for relative quantification.

### TdT‐Mediated dUTP Nick-End-Labeling Assay

TUNEL staining of the cardiac tissue was performed as described previously (Ye et al., 2020b). The sections of the cardiac tissue were analyzed using a TUNEL kit (Millipore, United States) following the manufacturer’s instructions. Light microscopy was used to evaluate apoptotic cells, and Image Pro Plus software was used for relative quantification.

### Western Blotting

We extracted and prepared total LV tissue protein with 1× RIPA buffer as reported previously (Ye et al., 2018). Proteins (50 μg) were separated by electrophoresis through a 10 or 12% sodium dodecyl sulfate polyacrylamide gel electrophoresis (SDS-PAGE) gel and transferred onto polyvinylidene fluoride membrane (IPFL00010, Millipore, Billerica, MA, United States). Then, the blots were blocked with 5% nonfat powdered milk and incubated overnight at 4°C with the primary antibodies. The primary antibodies used in this study were as follows: phospho-nuclear factor kappa-B p65 (p-P65, 1:500 dilution, Abcam, Cambridge, United Kingdom), nuclear factor kappa-B (NF-kB) p65 (T-P65, 1:1000 dilution, Abcam), NADPH oxidase 2 (Nox-2, 1:200, Santa Cruz, CA, United States), NADPH oxidase 4 (Nox-4, 1:200, Santa Cruz), nuclear factor-erythroid 2-related factor 2 (Nrf-2, 1:500, Gene Technology, Shanghai, China), heme oxygenase-1 (HO-1, 1:500, Gene Technology), glucose-regulated protein 78 (GRP78, 1:500 dilution, Cell Signaling Technology), phospho-protein kinase R-like ER kinase (p-PERK, 1:200, Santa Cruz), caspase 12 (1: 1,000 dilution, Cell Signaling Technology), phospho-eukaryotic initiation factor 2α (*p*-eIF2α, 1:1,000 dilution, Cell Signaling Technology), activating transcription factor 6α (ATF-6α, 1:200 dilution, Santa Cruz Biotechnology), C/EBP homologous protein (CHOP, 1:1,000 dilution, Cell Signaling Technology), cleaved caspase 3 (c-caspase 3, 1:500, Cell Signaling Technology), B cell lymphoma-2 (Bcl-2, 1:500, Abcam), BCL2-associated X (Bax, 1:500, Abcam), and glyceraldehyde-3-phosphate dehydrogenase (GAPDH, 1:1,000, Cell Signaling Technology). After several washes, the blots were incubated with the secondary antibody (goat anti-rabbit antibody) at a dilution of 1:5,000 to 1:10,000 for 1 h at 25°C in the dark. The membranes were sequentially washed several times in the dark and scanned using the Odyssey Imaging System (LI-COR Biosciences, Lincoln, United States), and the band intensities were measured.

### Real-Time Polymerase Chain Reaction Analysis

RNA was extracted from LV tissue using TRIzol (Invitrogen Life Technologies, Carlsbad, CA, United States). The concentration of RNA is measured by Nanodrop 2000 (Thermo Fisher Scientific, United States). The cDNA was synthesized from 2 µg of RNA from each group using oligo (DT) primers and the Transcriptor First Strand cDNA Synthesis Kit (Roche). RT+/RT-control was applied when cDNA was synthesized. Water without RNase and DNase was used to dilute DNA for three times before quantitative analysis. Quantitative analysis was conducted using a LightCycler 480 and SYBR Green Master Mix (Roche) follow the MIQE guidelines. The results were analyzed with the 2^−△△Ct^ method and normalized against GAPDH gene expression. Details of the primer sequences are presented in Supplementary Table S1.

### Statistical Analysis

Data were analyzed using SPSS 24.0 software and are presented as the mean ± standard deviation (SD). Comparisons between groups were made using one-way analysis of variance (ANOVA) followed by Tukey’s test. A *p* value <0.05 was considered statistically significant.

## Results

### RvD1 Ameliorates Cardiac Dysfunction in Mice Treated With Dox

**FIGURE 1:**
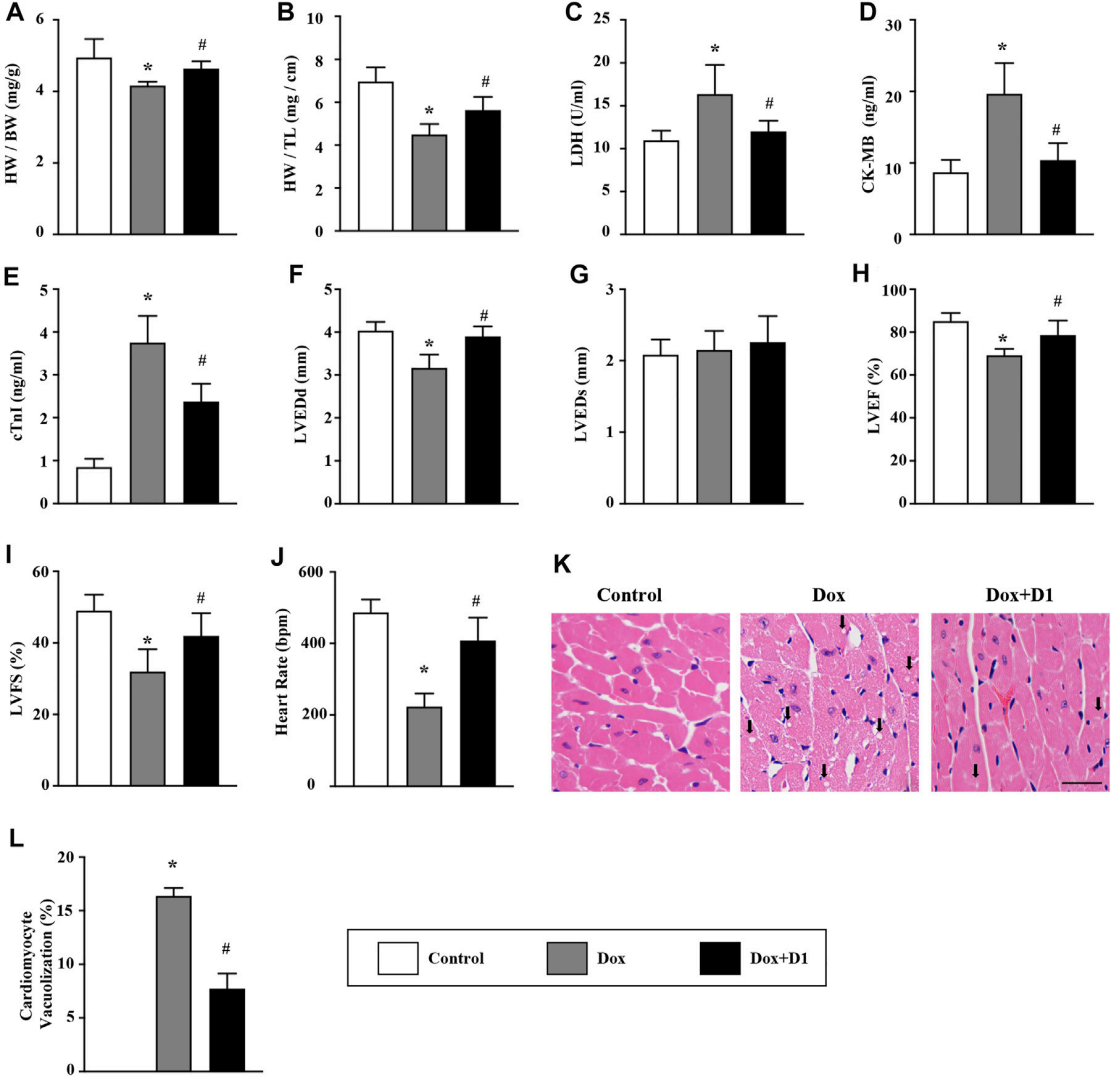
RvD1 improved cardiac function in mice treated with Dox. **(A, B)** The ratio of HW/BW and HW/TL in different groups, n = 10. **(C–E)** Level of LDH, CK-MB and cTnI in the serum, n = 5. **(F–J)** Echocardiographic parameters in different groups, including LVEDd, LVEDs, LVEF, LVFS and heart rate, n = 6. **(K, L)** Vacuolated cardiomyocytes were detected in different groups by H&E staining and quantified, n = 4, bar = 25 μm. Data was presented as the mean ± standard deviation (SD) and compared with one-way ANOVA followed by Tukey’s test. *p < 0.05 compared with the Control group. #*p* < 0.05 compared with the Dox group. HW, heart weight; BW, body weight; TL, tibia length; LDH, lactate dehydrogenase; CK-MB, myocardial-bound creatine kinase; cTnI, cardiac troponinwere I; LVEDd, left ventricular (LV) end-diastolic diameter; LVEDs, LV end-systolic diameter; LVEF, LV ejection fraction; LVFS, LV fractional shortening.

Compared to control mice, mice treated with Dox showed a decrease in the ratio of HW/BW and HW/TL. However, the administration of RvD1 improved the Dox-induced HW/BW and HW/TL ratios (Figures 1A,B). Compared to the control group, the levels of CK-MB, cTNI and activity of LDH in LV tissue were significantly increased in the Dox group. The administration of RvD1 reversed these trends (Figures 1C–E). In addition, the decreased LVEDd, LVEF, and LVFS in the Dox group were significantly improved by RvD1 treatment (Figures 1F–I). Histological examination revealed increased vacuolar accumulation in the Dox-treated mice, which was significantly improved in the Dox + RvD1 group (Figures 1J,K).

### RvD1 Reduces Dox-Induced Inflammation in Cardiac Tissue

**FIGURE 2:**
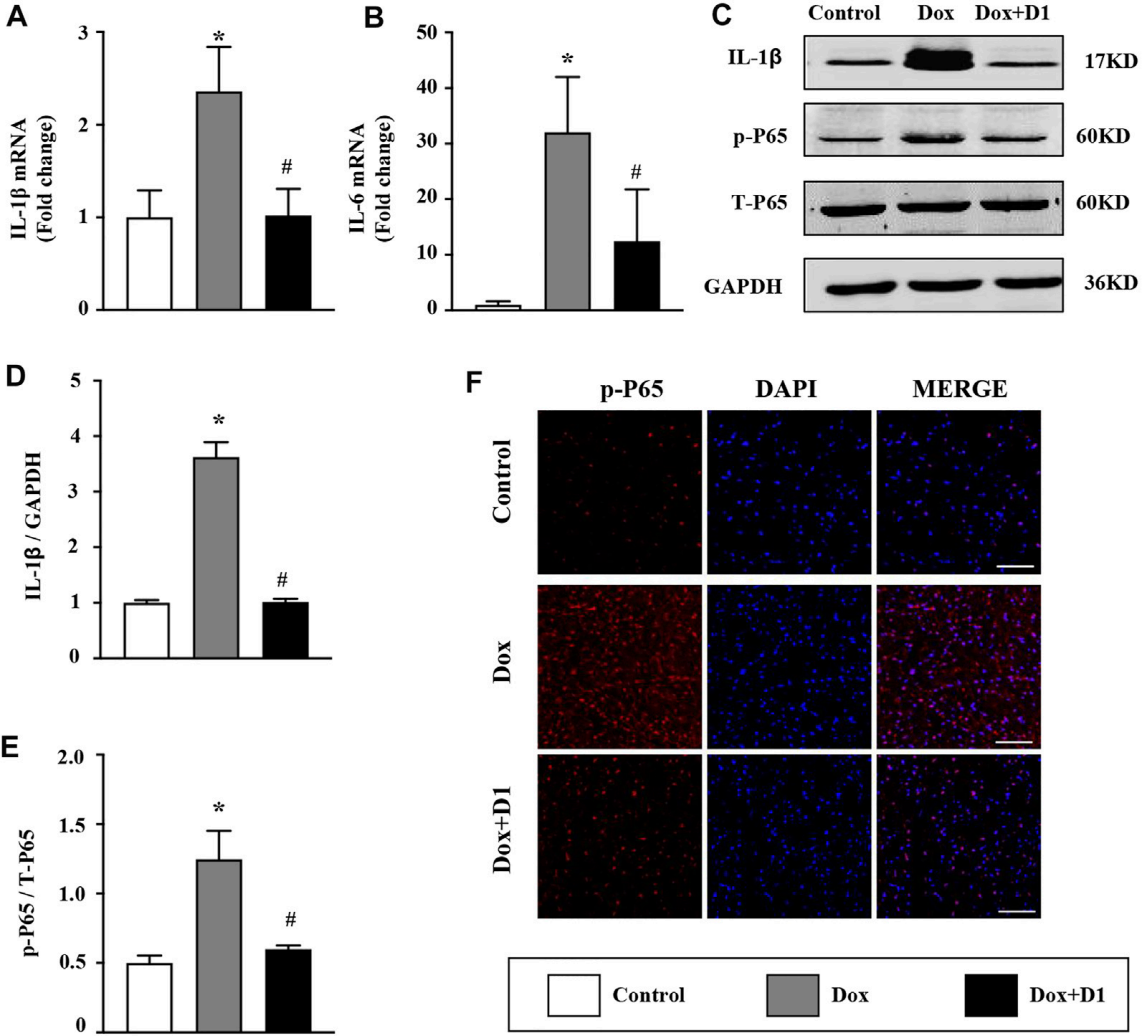
RvD1 reduced Dox-induced inflammation in cardiac tissue. **(A, B)** mRNA expression levels of the inflammatory cytokines, IL-1β and IL-6 in different groups, n = 4. **(C–E)** Western blotting of p-p65 in different groups, n = 4. **(F)** Immunofluorescence analysis of p65 in different groups, n = 4, bar = 50 μm. Data was presented as the mean ± SD and compared using one-way ANOVA followed by Tukey’s test. *p < 0.05 compared with the control group. #*p* < 0.05 compared with the Dox group.

The expression of proinflammatory cytokines including IL-1β and IL-6 in the heart was significantly increased by Dox treatment (Figures 2A,B). Compared to the Dox group, a significant reduction in IL-1β and IL-6 level was observed in the Dox + RvD1 group (Figures 2A,B). In addition, the inhibitory effect of RvD1 on inflammation was further confirmed by western blotting and immunofluorescence examination, which showed that RvD1 reduced NF-kB signaling (Figures 2C–E). These results showed that RvD1 protects against heart injury by inhibiting inflammatory responses.

### RvD1 Protects Cardiac Tissue Against Dox-Induced Oxidative Stress

**FIGURE 3:**
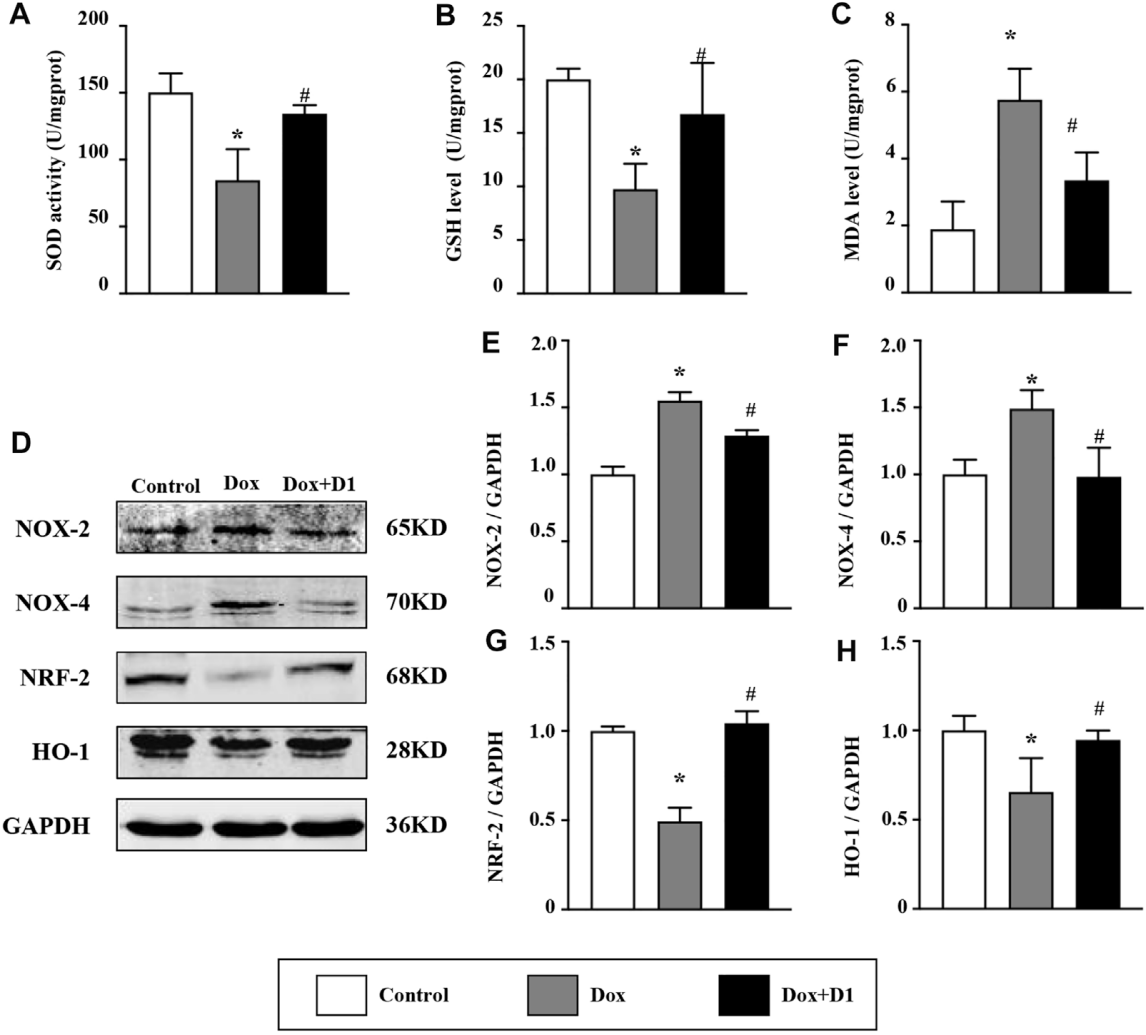
RvD1 protected the cardiac tissue against Dox-induced oxidative stress. Levels of superoxide dismutase (SOD) **(A)**, glutathione (GSH) **(B)** and malondialdehyde (MDA) **(C)** in left heart tissue in the three groups, n = 5. Representative western blotting **(D)** and results of quantitation of Nox-2 **(E)**, Nox-4 **(F)**, Nrf-2 **(G)** and HO-1 **(H)** in different groups, n = 4. Data was presented as the mean ± SD and compared using one-way ANOVA followed by Tukey’s test. *p < 0.05 compared with the control group. #*p* < 0.05 compared with the Dox group.

Compared to the control group, the activity of SOD and the expression level of GSH were reduced in the Dox group. Whereas, MDA level was significantly increased in the Dox group (Figures 3A–C). However, treatment with RvD1 significantly restored SOD activity and GSH level, and reduced MDA level compared to the Dox-treated group (Figures 3A–C). In addition, western blotting results showed that the expression of Nox-2 and -4, which are important generators of reactive oxygen species (ROS), was reduced in the Dox + RvD1 group compared to the Dox-treated group (Figures 3D–F). The expression of Nrf-2 and HO-1, which play protective roles against oxidative stress, was increased in the Dox + RvD1 group compared to the Dox group (Figures 3G,H). These findings indicate that RvD1 treatment protects the cardiac tissue against Dox-induced oxidative stress.

### RvD1 Attenuates Dox-Induced Endoplasmic Reticulum Stress

**FIGURE 4:**
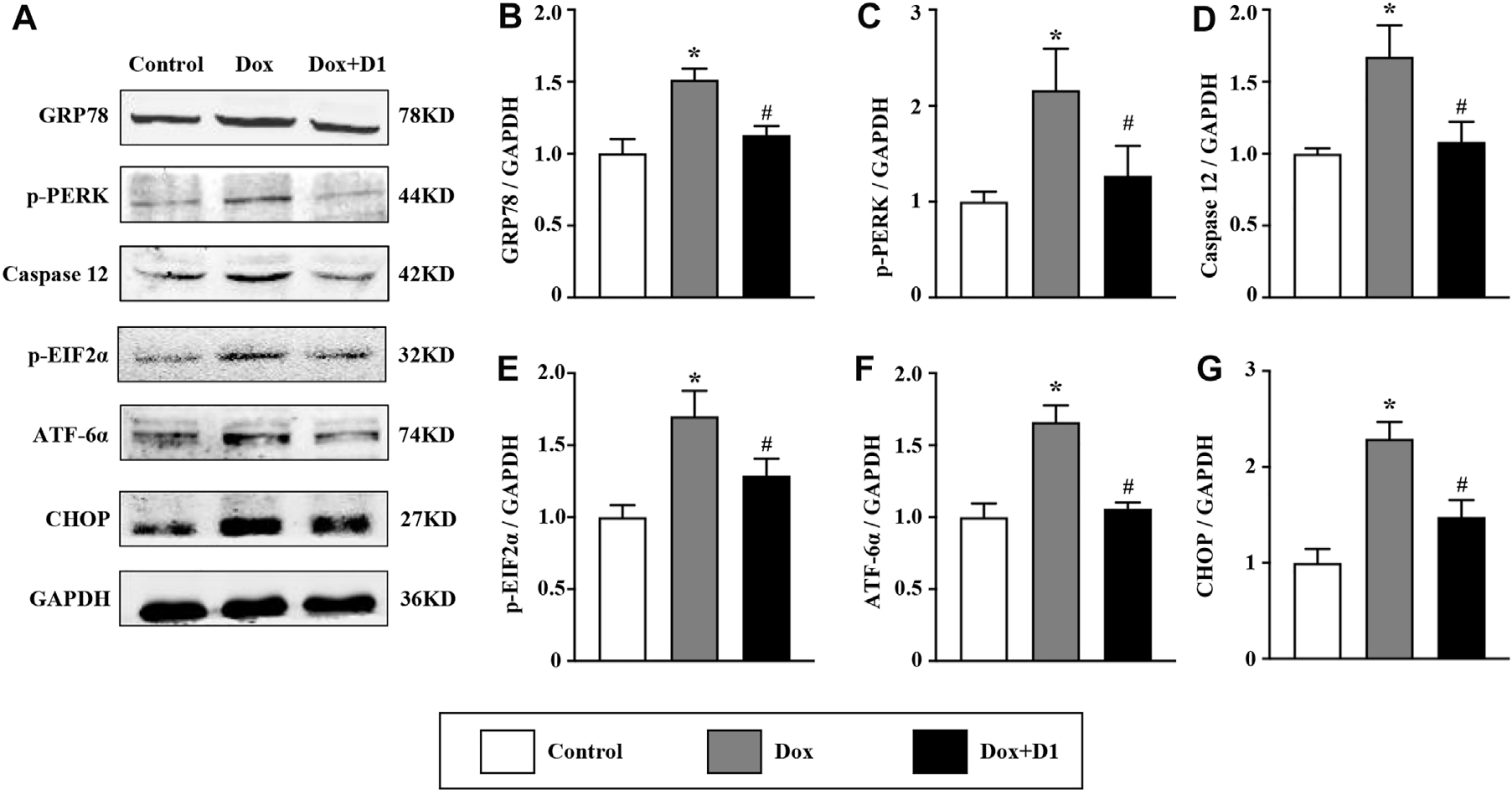
RvD1 attenuated Dox-induced ER stress. Representative western blotting **(A)** and quantitative analysis showing the expression levels of glucose-regulated protein 78 (GRP78) **(B)**, protein kinase R-like ER kinase (PERK) **(C)**, caspase 12 **(D)**, phosphorylated eukaryotic translation initiation factor 2 (p-eif2α) **(E)**, activating transcription factor 6α (ATF6α) **(F)**, and C/EBP homologous protein (CHOP) **(G)** in different groups, n = 4. Data are presented as the mean ± SD and compared using one-way ANOVA followed by Tukey’s test. *p < 0.05 compared with the control group. #*p* < 0.05 compared with the Dox group.

We investigated whether RvD1 attenuated ER stress, thereby reducing cardiotoxicity induced by Dox. The western blot results showed that RvD1 treatment reduced the expression of important markers that are indicative of the severity of ER stress (Figure 4A), including GRP78 and CHOP (Figures 4B,G). In addition, the administration of Dox increased the expression levels of caspase-12, p-PERK, p-eif2α, and ATF6α, which were attenuated by RvD1 treatment (Figures 4C–F). These results indicate that RvD1 treatment attenuated Dox-induced ER stress.

### RvD1 Reduces Dox-Induced Apoptosis of Cardiomyocytes

**FIGURE 5:**
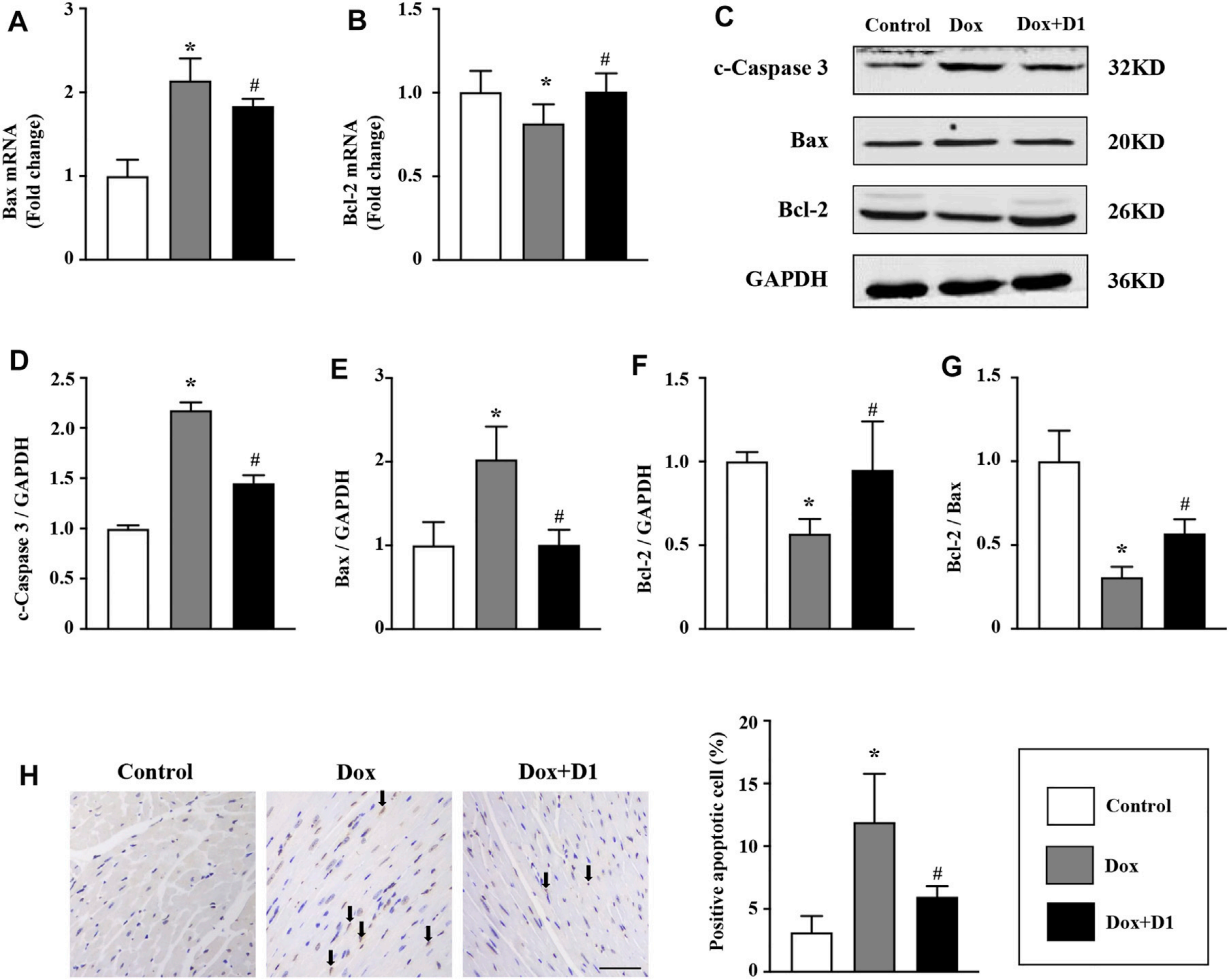
RvD1 reduced Dox-induced apoptosis of cardiomyocytes. mRNA levels of Bax **(A)** and Bcl-2 **(B)** in different groups, n = 4. Representative western blotting **(C)** and quantitative analysis showing the expression levels of c-caspase-3 **(D)**, Bax **(E)**, and Bcl-2 **(F)** in different groups, n = 4. **(G)** Bcl-2/Bax in different groups. **(H)** TdT‐mediated dUTP nick-end-labeling (TUNEL) assay results of the left cardiac tissue in different groups, n = 4, bar = 50 μm. Data are presented as the mean ± SD and compared using one-way ANOVA followed by Tukey’s test. *p < 0.05 compared with the control group. #*p* < 0.05 compared with the Dox group.

Apoptosis is reported to be involved in Dox-induced cardiotoxicity (Wang et al., 2016a). We evaluated the severity of apoptosis and identified the potential signaling pathways associated with the RvD1 treatment on cardiomyocyte apoptosis. Compared to the control group, the mRNA level of Bax was increased while that of Bcl-2 was decreased in the Dox-treated group (Figures 5A,B). These changes were significantly reversed following treatment with RvD1. Similarly, western blotting results showed that Dox increased the expression levels of c-caspase 3 and Bax, and decreased Bcl-2 expression (Figures 5C–G). These changes were attenuated by RvD1. In addition, TUNEL assay showed that RvD1 treatment significantly reduced the number of Dox-induced apoptotic cells (Figure 5H). These results showed that RvD1 reduces Dox-induced cardiomyocyte apoptosis.

## Discussion

This study revealed that RvD1 attenuates Dox-induced cardiotoxicity. The possible mechanisms involved in RvD1-mediated attenuation of Dox-induced cardiotoxicity include regulation of inflammation, oxidative stress, autophagy dysfunction, ER stress, and apoptosis.

Dose-dependent cardiac toxicity and heart failure were reported in cancer patients treated with Dox, which seriously impaired the quality of life and life expectancy of patients with cancer (VON HOFF et al., 1979). Dox triggers splenic contraction and irreversible dysregulation of cyclooxygenase and lipoxygenase, which alter the inflammation resolution program in the myocardium, suggesting that resolvin supplementation may improve the cardiac injury induced by Dox (Jadapalli et al., 2018). RvD1, an important anti-inflammatory mediator, is mainly derived from DHA. DHA supplementation may attenuate Dox-induced cardiotoxicity by inhibiting the activation of NF-κB/iNOS/NO signaling pathway *in vitro* (Wang et al., 2016b). In addition, another study suggested that DHA pretreatment may protect H9C2 cells against Dox-induced injury by reducing ROS production (Hsu et al., 2014). However, the *in vivo* effects of RvD1 on Dox-induced cardiotoxicity remain unknown. In this study, RvD1 treatment increased the HW/BW ratio, reduced the level of cardiac injury biomarkers, and improved the EF and FS, all of which were compromised by Dox. Taken together, RvD1 ameliorated cardiac dysfunction in mice treated with Dox.

Inflammation is the body’s defensive response to stimulation, such as infection or injury. Acute inflammatory responses such as surgery-induced tissue injury are self-limited processes that resolve on their own and are divided into initiation and resolution phases. Failed inflammation resolution can lead to immunopathology, such as systemic inflammation leading to organ dysfunction and death. Increasing evidence shows that polyunsaturated fatty acid-derived lipid mediators such as lipoxin A4, resolvins, and protectins are produced during the onset of inflammatory reactions. Their important biological roles have been demonstrated in a variety of cell types *in vitro* and in many animal models of diseases *in vivo* (Takano et al., 1997; Takano et al., 1998; Serhan and Petasis, 2011). Dox is reported to induce inflammatory responses through enhanced expression and release of proinflammatory cytokines by activating the NF-kB signaling pathway in the heart (Pecoraro et al., 2016). In the present study, we showed that Dox treatment provoked a series of inflammatory responses and increased the expression of inflammatory cytokines. The administration of RvD1 significantly reduced the expression of proinflammatory cytokines, including IL-1β and IL-6, by suppressing the NF-kB signaling pathway. This study indicates that RvD1 confers a potential cardioprotective effect against Dox-induced cardiotoxicity through the inhibition of the inflammatory response.

ROS are generated during mitochondrial oxidative metabolism and in cellular response to xenobiotics, cytokines, and bacterial invasion (Ray et al., 2012). When ROS overwhelms the cellular antioxidant defense system, whether through increased levels of ROS or decreased cellular antioxidant capacity, oxidative stress occurs (Ray et al., 2012). RvD1 was reported to suppress oxidative stress by upregulating the expression of Nrf2 and HO-1 in other diseases (Saito et al., 2018). In the present study, lipid peroxidation products (MDA) and antioxidant enzymes (SOD and GSH) were used to estimate the oxidative stress in the cardiac tissues. Administration of RvD1 significantly attenuated the oxidative stress induced by Dox treatment. Dox induces the production of ROS through activation of the NADPH oxidase signaling pathway (Wojnowski et al., 2005). Nox-2 deficiency protects mice against cardiac injury after Dox treatment (Zhao et al., 2010). In this study, we found that RvD1 treatment inhibited oxidative stress by downregulating the expression of Nox-2 and Nox-4, the key NADPH oxidase subunit. In addition, ER stress plays an important role in the development of heart failure (Minamino et al., 2010). ER stress establishes a progressive pathological cycle with oxidative stress in endothelial dysfunction, diabetes, and heart, liver, kidney, or neurological diseases (Reyes-Fermín et al., 2020). ER stress plays an important role in oxidative stress, as it is also a source of ROS (Burgos-Morón et al., 2019). We evaluated the level of ER stress to further understand the underlying mechanisms behind RvD1-mediated protection against Dox-induced cardiotoxicity. ER stress-associated proteins were enhanced following Dox treatment but reduced after RvD1 administration. The RvD1-mediated protection against Dox-induced ER stress was achieved by downregulating the PERK and ATF-6 signaling pathways. These results indicate that RvD1 may suppress Dox-induced cardiotoxicity through the regulation of oxidative stress and ER stress.

Several studies have indicated that Dox promotes endoplasmic reticulum-induced apoptosis by activating the expression of pro-apoptotic factors and inhibiting the expression of anti-apoptotic factors (Chua et al., 2009). As a specific pro-apoptotic pathway, ER stress can activate the CHOP and caspase-12 pathways, thereby mediating apoptosis (Tabas and Ron, 2011). In the present study, RvD1 treatment reduced the expression of CHOP and caspase-12, leading to reduced myocardial apoptosis and improved cardiac dysfunction. In addition, CHOP is reported to regulate apoptosis factors, including Bax, Bcl-2, and cleaved caspase-3, which are key determinants of cell death (Fu et al., 2010). Consistent with previous studies, our results showed that RvD1 treatment significantly enhanced the expression of Bcl-2 and reduced the expression of Bax and cleaved caspase-3. This study demonstrates that RvD1 protects cardiac tissues against Dox-induced apoptosis.

Our study has several limitations. First, the administeration of RvD1 before Dox may prevent the absorption of Dox into systemic circulation due to chemicophysical interaction. Second, our study suggests that RvD1 may improve Dox-induced cardiotoxicity via alleviating apoptosis. Further interventions may help to explore the mechanism. Third, we did not detect the systemic inflammation induced by Dox in this study.

## Conclusion

**FIGURE 6:**
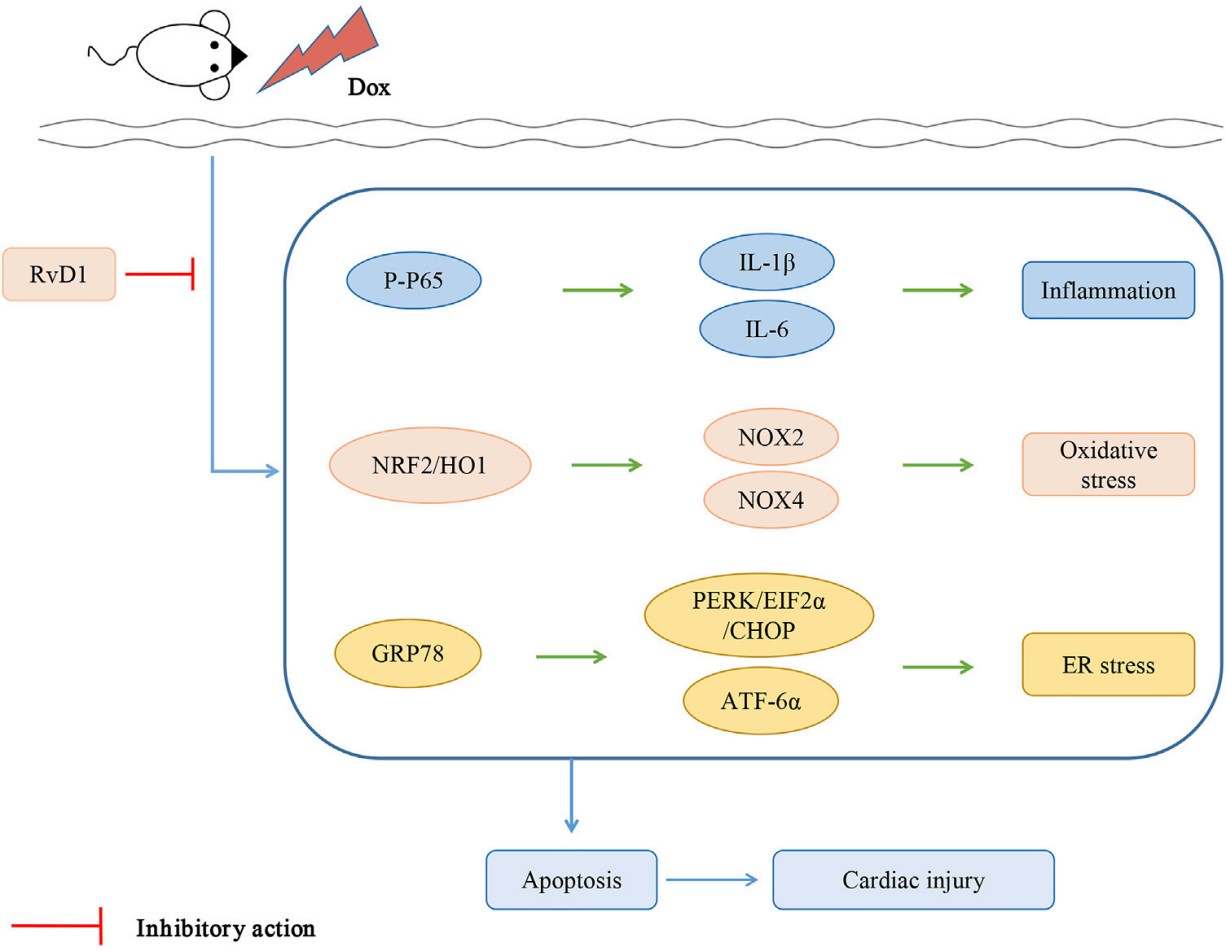
Diagram depicting the regulation mechanism of RvD1 in Dox-induced cardiotoxicity.

RvD1 protects cardiac tissue against Dox-induced cardiotoxicity, possibly through the attenuation of inflammation, oxidative stress and ER stress (Figure 6).

## Data Availability

The raw data supporting the conclusions of this article will be made available by the authors, without undue reservation.
