# Supplementary material for: Resolvin D1 Attenuates Doxorubicin-Induced Cardiotoxicity by Inhibiting Inflammation, Oxidative and Endoplasmic Reticulum Stress
Source: Front Pharmacol. 2022 Jan 5;12:749899. doi: 10.3389/fphar.2021.749899 (PMC8769281; doi:10.3389/fphar.2021.749899)
Supplement: Supplementary file 1 [file DataSheet1.docx]

TABLE 1: Primers for quantitative real-time PCR.

| Gene | Forward primer (5′-3′) | Reverse primer (5′-3′) |
| --- | --- | --- |
| IL-1β | GGGCCTCAAAGGAAAGAATC | TACCAGTTGGGGAACTCTGC |
| IL-6 | CCAAGAGGTGAGTGCTTCCC | CTGTTGTTCAGACTCTCTCCCT |
| Bax | TGAGCGAGTGTCTCCGGCGAAT | GCACTTTAGTGCACAGGGCCTTG |
| Bcl-2 | TGGTGGACAACATCGCCCTGTG | GGTCGCATGCTGGGGCCATATA |
| GAPDH | AACTTTGGCATTGTGGAAGG | CACATTGGGGGTAGGAACAC |
